# The scent of death: A case study for volatile markers of decomposition on a concrete floor

**DOI:** 10.1111/1556-4029.70271

**Published:** 2026-01-28

**Authors:** Alexis Hecker, Anna Painter, John Goodpaster

**Affiliations:** ^1^ Forensic and Investigative Sciences Program, Department of Chemistry and Chemical Biology Indiana University Indianapolis Indianapolis Indiana USA; ^2^ Michigan City Police Department Michigan City Indiana USA

**Keywords:** concrete substrate, decomposition, gas chromatography–mass spectrometry (GC–MS), solid phase microextraction (SPME), volatile organic compounds (VOCs)

## Abstract

Volatile organic compounds (VOCs) released during human decomposition are chemically diverse and can provide forensic evidence indicating the prior presence of a corpse. In July 2023, the Michigan City Police Department received a report from an individual claiming to have murdered his roommate and stored the body in a basement cellar for 57 days before dismemberment and disposal. Concrete core samples from the basement were analyzed using headspace solid‐phase microextraction coupled with gas chromatography–mass spectrometry (HS‐SPME GC–MS). Three concrete samples contained six VOCs that are known to originate from the decomposition process. This leads to the conclusion that the decomposing body of someone or something was present in the room for enough time for the decomposition VOCs to collect on, in, and under the floor. This case represents the first successful legal introduction of VOC analysis evidence from concrete substrates within the State of Indiana.

**Highlights**

- HS‐SPME‐GC–MS detected decomposition VOCs in basement concrete cores.
- Six decomposition VOCs indicated prolonged presence of decomposing remains.
- Concrete's porosity allowed VOC accumulation on, within, and beneath the floor.
- First Indiana case admitting concrete VOC evidence under Daubert standards.

## 1 INTRODUCTION

Human decomposition involves complex biochemical processes, including autolysis and putrefaction, that produce numerous volatile organic compounds (VOCs). Autolysis, driven by the body's own enzymes, initiates cellular breakdown, whereas putrefaction, mediated by microbial activity, generates gases and distinctive odorants. The chemical diversity of decomposition VOCs includes short‐chain fatty acids, ketones, aldehydes, alcohols, sulfur‐containing compounds, and various nitrogenous compounds [1, 2, 3, 4, 5, 6]. These VOCs result from enzymatic hydrolysis, bacterial metabolism of proteins, lipids, and carbohydrates, and oxidative processes [1, 7]. Specific compounds like short‐chain fatty acids and ketones arise primarily from lipid degradation [8]. Importantly, current forensic methodologies detect these decomposition VOCs but cannot differentiate between human and animal origins, which presents limitations in forensic interpretation [2, 9].

Extensive research has documented common VOC profiles associated with human remains, although definitive human‐specific markers remain elusive [10, 11, 12]. Despite this limitation, detection of these VOCs can strongly corroborate investigative hypotheses involving decomposition events. This study utilizes headspace solid‐phase microextraction coupled with gas chromatography–mass spectrometry (HS‐SPME GC–MS) to identify decomposition VOCs in concrete samples, providing forensic insight into claims of human remains being stored at a crime scene. Previous forensic VOC studies have primarily utilized soil or fabric substrates, making this concrete analysis particularly novel.

## 2 CASE HISTORY

On August 29, 2022, a man by the name of John Hallett contacted police agencies in both Bellingham, Washington, and Michigan City, Indiana. He wished to confess to the murder and dismemberment of his roommate, Paul Gonzales, on November 25, 2017, in Michigan City, Indiana. The story provided to law enforcement was that John Hallett suspected Gonzales of stealing Hallett's mail and conspiring to have him evicted. Gonzales, who was in his 60s, had muscular dystrophy. He required a cane to walk and received regular assistance from service providers due to his disability. Hallett attacked Gonzales with a crutch in the early hours of the morning as Gonzales was leaving the bathroom. After Gonzales fell to the ground, Hallett repeatedly hit Gonzales with the crutch. When Gonzales grabbed the crutch during the attack, Hallett punched Gonzales repeatedly in the face until he was unconscious. Then, Hallett strangled him. He kept his body in a bedroom for 4 days and then moved the body to the basement of the home. For 56 days, Hallett kept Gonzales's decomposing body in the basement. Hallett then used a hacksaw to dismember Gonzales's body into multiple pieces before placing these body parts in construction‐type heavy‐duty trash bags. He triple‐bagged each body section and left them for the regular garbage collection. Hallett then cleaned the basement floor with a wire brush and abrasives. He also mopped and painted the basement floor repeatedly to conceal the evidence left by Gonzales's decomposing body. The body was never found.

This confession led to a unique investigation in which there was no missing person report and no contact with friends and family by Paul since 2017. Det. Lt. Anna Painter and Det. Cpl. Kay Pliske conducted an investigation in which they learned that Paul was still receiving Social Security payments with no withdrawals from his account since November 23, 2017. His cellular phone was turned off for non‐payment in 2017. He was evicted from the residence he shared with John Hallett for non‐payment and failed to respond to the eviction notice. Paul was frail and being treated for a debilitating illness but was not refilling any of his prescription medications. None of Paul's caretakers had any contact with him after November 25, 2017, and had in fact called in two separate welfare checks on him. On one of those welfare checks, John Hallett told police that Paul left with his girlfriend.

**FIGURE 1:**
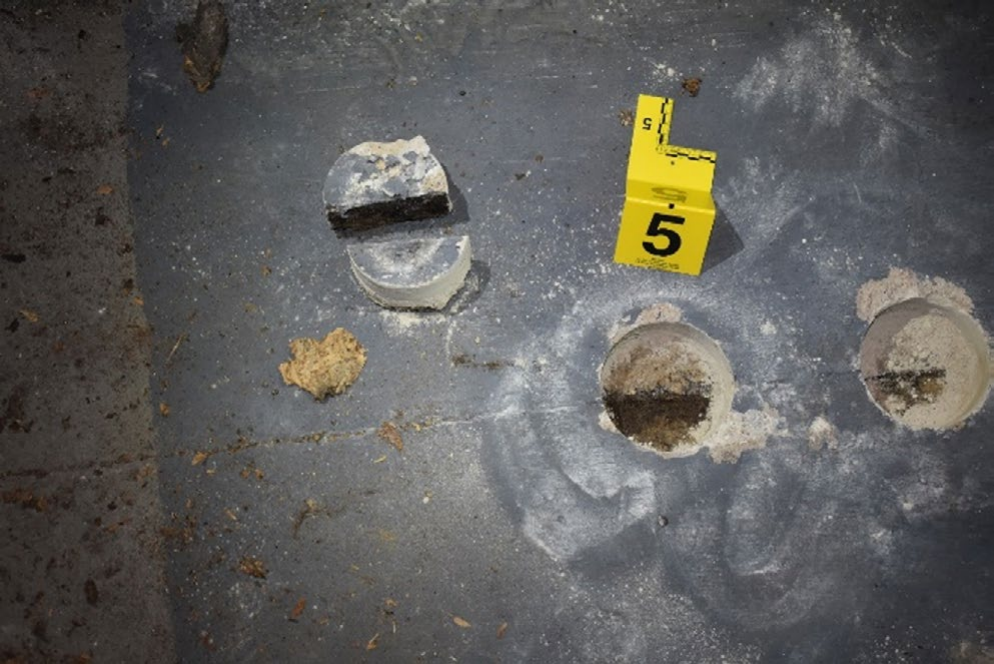
Photo of concrete core samples taken by Michigan City Police Department.

**FIGURE 2:**
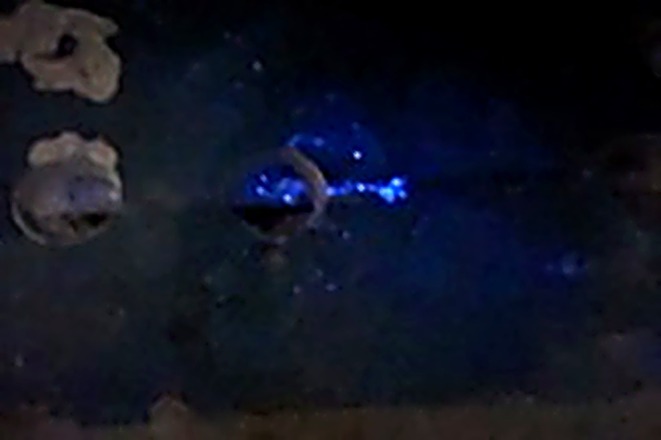
Photo of positive BlueStar® presumptive test taken by Michigan City Police Department.

With Paul's digital footprint disappearing on November 23, 2017, there was reason to believe that John Hallett's confession might be true. The next step was to bring in a cadaver canine to search the basement where John told investigators he kept Paul for 56 days. Cadaver canines serve as presumptive biological detection tools, specially trained to identify human decomposition scent and alert their handler to its location. The cadaver canine alerted to the odor of decomposition in the room where Paul was to have been kept. Investigators noted that the painted concrete floor had visible seams, which are potential collection points for bodily fluids (Figure 1). Presumptive testing using BlueStar® indicated positive results for blood (Figure 2).

The investigators then carried out an additional search of the residence in which core samples were drilled out of the basement floor for potential DNA/VOC testing. However, the core samples were found to be unsuitable for DNA testing. Then investigators saw a news report about the murder of Kristin Smart being solved from soil testing for decomposition. This led the investigators to research the type of testing used in the Kristin Smart case, which ultimately led to Det. Lt. Painter reaching out to Professor Goodpaster at IUI who agreed to test the core samples.

Murder convictions where the victim's body has NOT been recovered are rare but not unprecedented. In the United States, hundreds of such cases exist but they require strong circumstantial and forensic evidence. A notable example is Kristen Smart, who disappeared in 1996. Paul Flores was convicted of her murder in 2022. A search of his father's home located a 6‐foot by 4‐foot anomaly in the soil under his deck, and testing confirmed the presence of human blood. Another related case would be that of Casey Anthony, who was accused of killing her 2‐year‐old daughter, Caylee. Skeletal remains of the young girl were later found. Expert testimony stated that the strong odor emanating from the trunk of Casey's car could only be produced by organic decomposition. Ms. Anthony was ultimately acquitted.

## 3 MATERIALS AND METHODS

A total of seven concrete core samples were collected from the basement floor by the Michigan City Police. Items #4–6 served as comparison samples, while Items #8–11 were collected from areas identified as likely decomposition sites based on prior examinations and Bluestar‐positive results (Table 1).

**TABLE 1:** Item number assigned to the corresponding sample provided by Michigan City Police Department.

Item #	Sample description
4	Comparison #1
5	Comparison #2
6	Comparison #3
8	Room Sample #5
9	Room Sample #6
10	Room Sample #7
11	Room Sample #8

**FIGURE 3:**
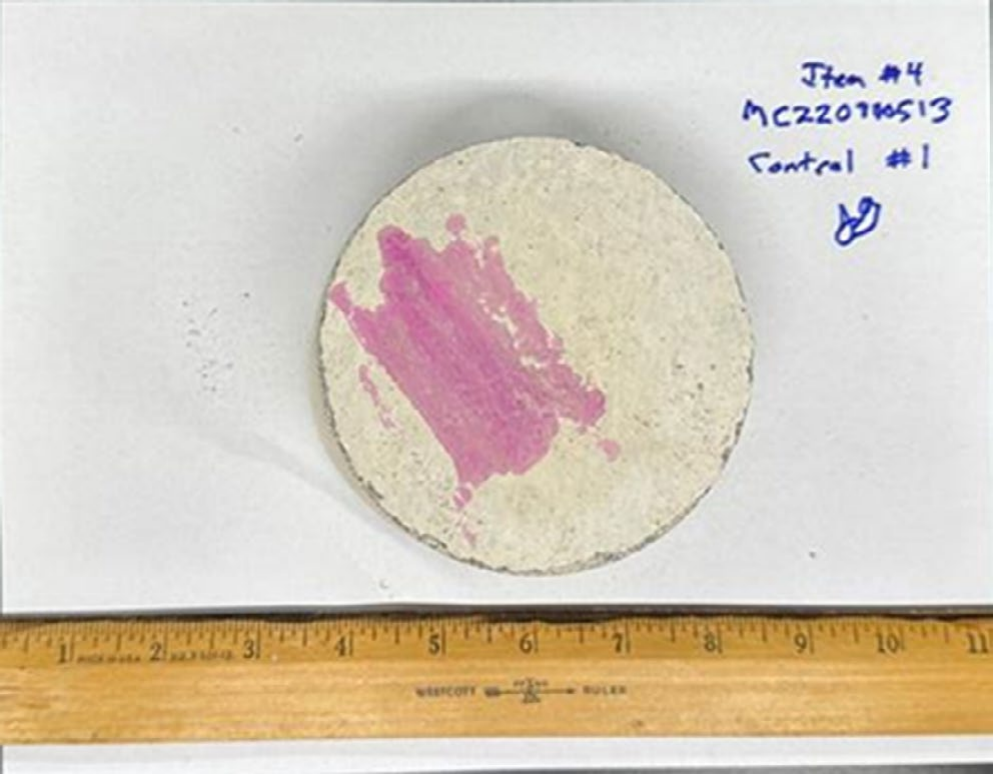
Photo of the top of item #4, a concrete core sample sprayed with BlueStar®.

**FIGURE 4:**
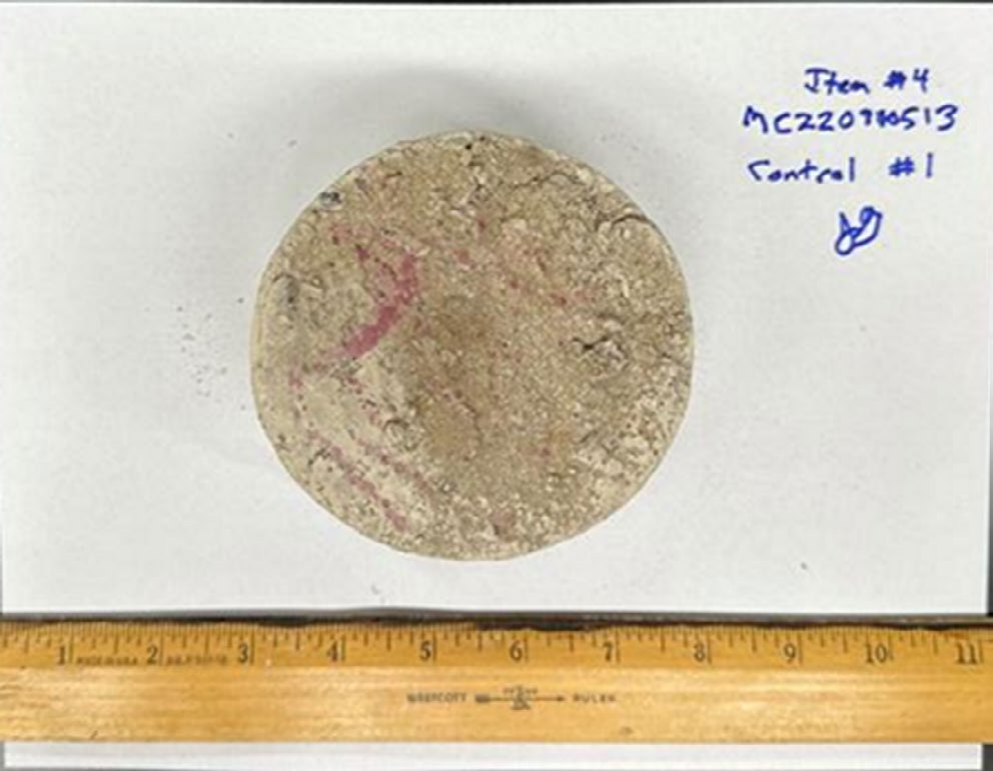
Photo of the bottom of item #4, a concrete core sample.

Each item was individually placed on a clean sheet of white paper and photographed from multiple angles to document condition and surface details (Figures 3 and 4).

Loose soil and debris were carefully scraped with a clean spatula and stored separately in 20 mL screw‐top headspace vials. Paint chips were removed with clean forceps and stored separately in labeled headspace vials. The concrete surfaces were swabbed using a two‐step method: First with a cotton swab wetted with HPLC grade water, followed immediately by a dry cotton swab. Both wet and dry swabs were separately stored in labeled headspace vials. HS‐SPME GC–MS analysis was performed on each sample vial. System blanks, HPLC grade water controls, concrete scraping controls, paint chip controls, and dry and wet swab controls were analyzed to account for potential contamination. Compound identification relied on matching mass spectra with an extensive spectral library (NIST 11). Peaks were scored numerically, with values above 80 considered significant matches.

An Agilent 6890 GC coupled to an Agilent 5975 Mass Selective Detector with an attached Gerstel multipurpose sampler was used for all experiments. The GC column was an Agilent Technologies HP‐5MS Ultra Inert column with a length of 30 m, a 0.250 mm inner diameter, and a 0.25‐μm‐film thickness. Tapered inlet liners of 2.0 mm inner diameter from Restek and 100 um polydimethylsiloxane (PDMS) from Supelco were used for SPME analyses.

The incubation temperature was 40°C and the incubation time was 1 min. The sample extraction and desorption times were 30 min and 60 s, respectively. The inlet temperature was set to 250°C and was operated with a 20:1 split ratio. The initial oven temperature was 40°C and was held for 1 min, then the temperature was ramped at 15°C/min to 300°C where it was held for 1 min. The SPME fiber was baked out at 250°C for 2 min preinjection and 1 min postinjection. The transfer line was set to 280°C. The quadrupoles were kept at 150°C and the source was kept at 230°C. A scan range of *m*/*z* 40–*m*/*z* 550 was used, with no solvent delay.

**FIGURE 5:**
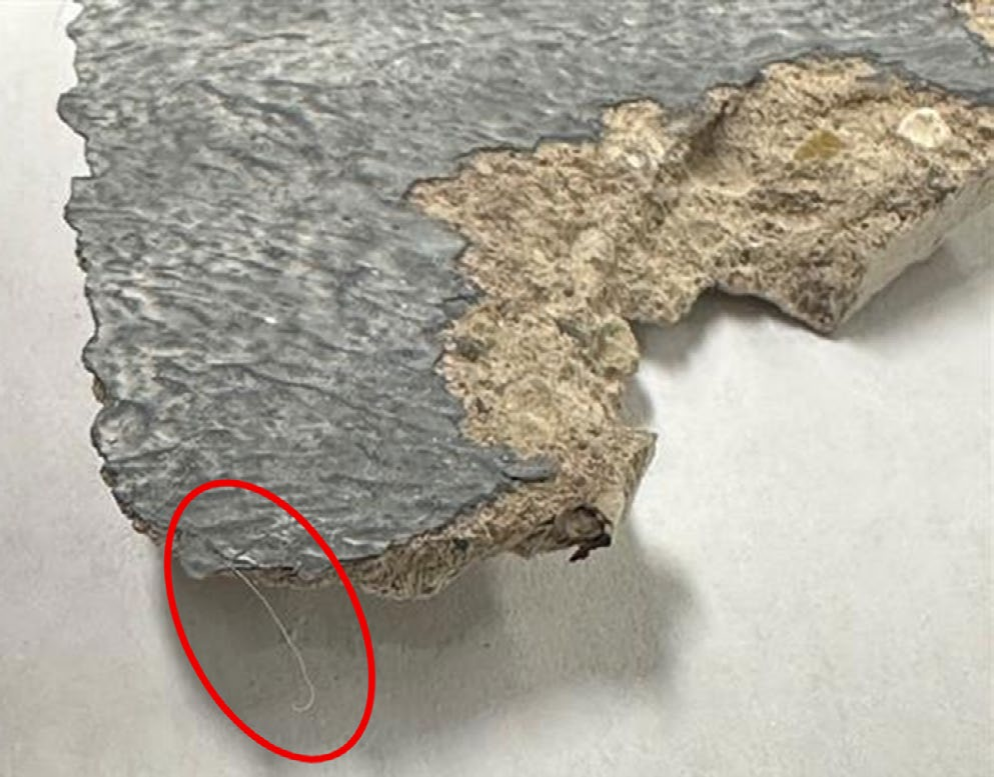
Photo of possible hair fiber (circled in red) collected from item #10.

During visual inspection, three hair/fiber samples embedded in paint layers of the concrete samples were separately collected, stored in Ziploc bags, and recommended for microscopic and genetic analysis (Figure 5).

## 4 RESULTS

**FIGURE 6:**
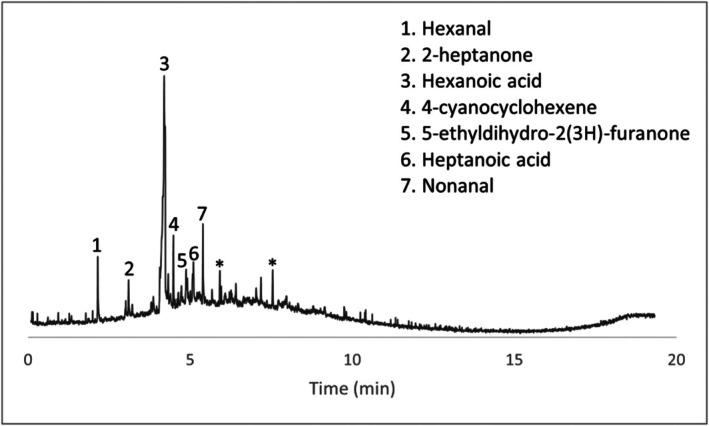
Chromatogram of wet Q‐tip control.

Several compounds were identified in the wet swab negative control (Figure 6). Peaks labeled with an asterisk are peaks seen in the blanks and peaks that are not labeled were unable to be identified with confidence. Given that the HPLC grade water control did not produce any VOCs, these may have originated from extractables from the swab itself. Two of these specific compounds, hexanoic acid and nonanal, were also seen in comparison samples (items #4 and #5) as well as in peer‐reviewed articles on decomposition volatiles. In general, if either of these compounds appeared in actual wet swab samples, they were discounted. Concrete scraping controls, paint chip controls, and dry swab controls presented no decomposition‐related VOCs.

**FIGURE 7:**
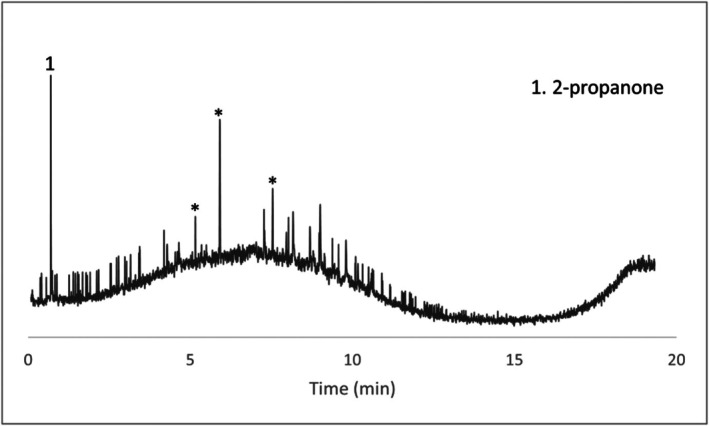
Chromatogram of scraping from Item #8.

**FIGURE 8:**
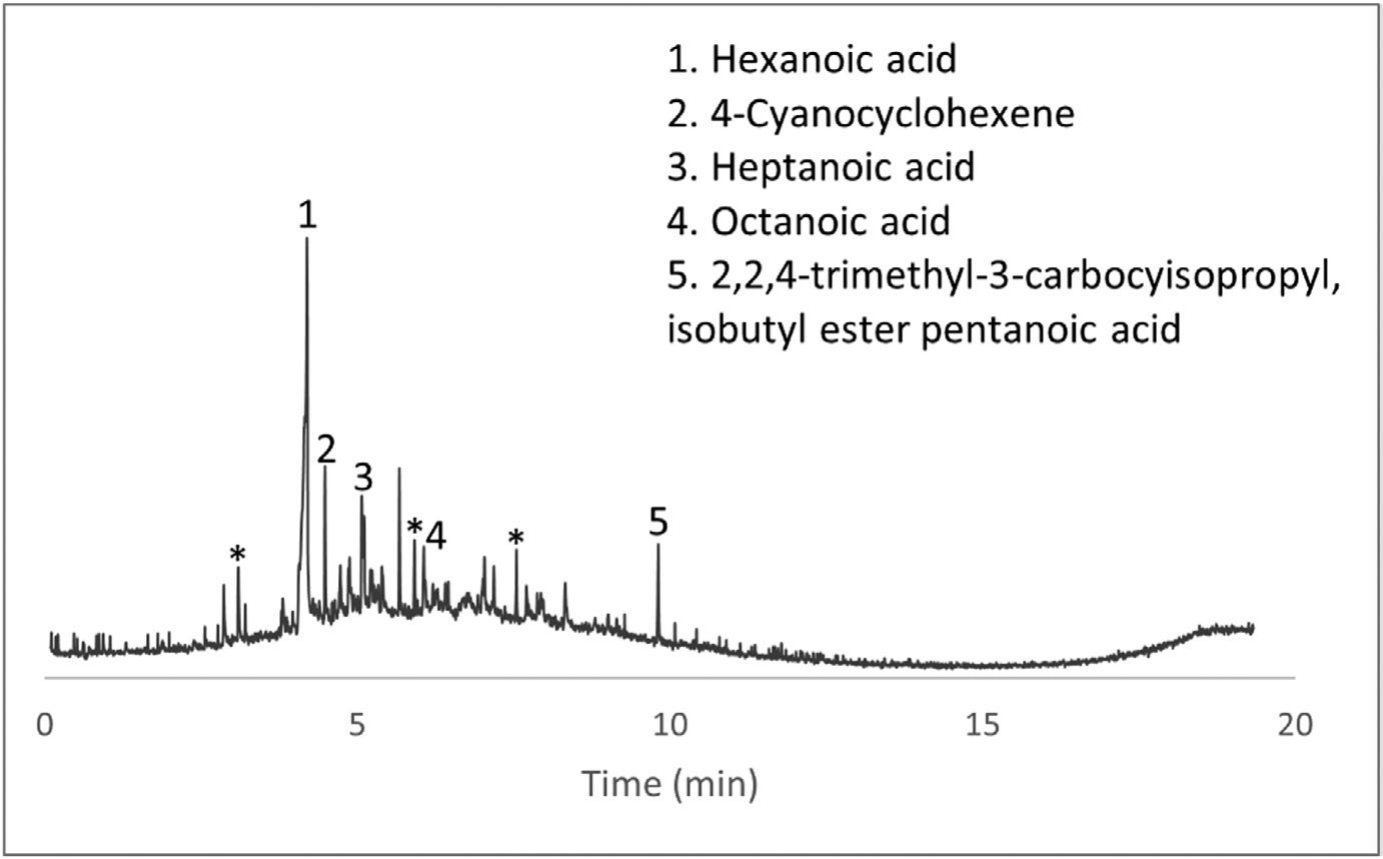
Chromatogram of wet swab from Item #8.

**FIGURE 9:**
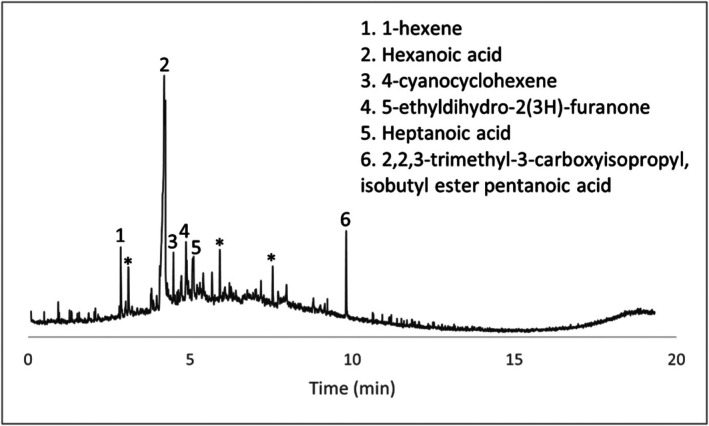
Chromatogram of wet swab from Item #9.

**FIGURE 10:**
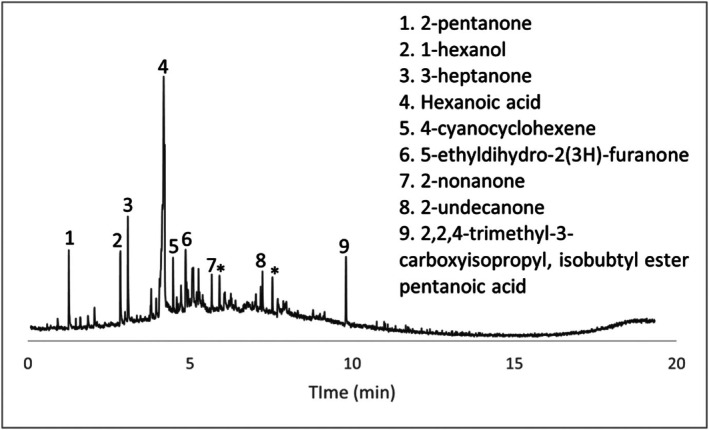
Chromatogram of wet swab from Item #10.

Concrete samples and their associated chromatograms revealed several characteristic decomposition VOCs. Items #8, #9, and #10 provided chromatographic evidence (Figures 7, 8, 9, 10).

All VOCs detected across the samples are summarized in Table S1. VOCs associated with decomposition are listed below, along with their corresponding samples and potential sources (Table 2).

**TABLE 2:** Items containing at least one detected decomposition VOC and the known sources of those VOCs.

Item #	Description	Decomposition VOCs	Compound class	Tissue type	References
6	Comparison #3 (scraping)	1‐propanol	Alcohol	Human Pig	[6, 12, 13, 14]
		1‐butanol	Alcohol	Human Pig	[2, 6, 12, 13, 14, 15, 16]
8	Sample #5 (scraping)	2‐propanone (acetone)	Ketone	Human Pig	[2, 12, 16, 17, 18]
8	Sample #5 (wet swab)	Octanoic acid	Aliphatic fatty acid	Pig	[16, 18]
9	Sample #6 (wet swab)	1‐hexene	Alkene	Human pig	[2, 6, 18]
10	Sample #7 (wet swab)	2‐pentanone	Ketone	Human Pig	[2, 12, 14, 15, 19]
		2‐nonanone	Ketone	Human Pig Chicken	[6, 13, 15, 19, 20]
		2‐undecanone	Ketone	Human Pig Chicken	[6, 13, 15, 19, 20]

The analysis indicates that three concrete samples contained six VOCs known to originate from the decomposition process. These findings suggest that a decomposing body, either human or animal, was present in the room long enough for decomposition‐related VOCs to accumulate on, within, and beneath the floor.

Additionally, scraping from Comparison #3 (Item #6) also contained two decomposition‐related VOCs. This result was not unexpected, given that the comparison samples were collected from the same room as the suspected samples (Items #8–11).

## 5 DISCUSSION

The compound classes identified and detailed in Table 2 align with known biochemical pathways of decomposition. Alcohols are commonly produced through microbial fermentation of carbohydrates and amino acids as well as by reduction of aldehydes and ketones during early putrefaction [2, 13, 14]. Ketones such as 2‐propanone and 2‐nonanone arise from lipid oxidation and β‐oxidation of fatty acids, processes that dominate as soft tissue fats and cell membranes degrade [2, 15, 16, 17]. Aliphatic fatty acids, including volatile fatty acids, are generated via hydrolysis of triglycerides and microbial fermentation of lipids and proteins, often accompanied by saponification under moist, anaerobic conditions [2]. Alkenes and other hydrocarbons result from oxygen‐free‐radical‐mediated lipid peroxidation of polyunsaturated fatty acids, a late‐stage oxidative process observed in both human and animal decomposition [2, 13, 18]. Together, these pathways explain the presence of alcohols, ketones, fatty acids, and alkenes among the detected VOCs and support their origin from decomposition processes described in prior studies [2, 11, 19].

The detection of two decomposition VOCs in Comparison #3 (Item #6) likely reflects the physical spread of bodily fluids and the diffusion of the decomposition VOCs within the same room. Given the porous nature of concrete, it is possible that bodily fluids initially spread across the surface of the floor and subsequently seeped into the material, allowing decomposition‐related compounds to penetrate and accumulate in nearby areas where the comparison samples were collected. In addition, VOCs are inherently volatile and mobile in enclosed environments, allowing them to evaporate, diffuse through air, and adsorb onto nearby surfaces such as flooring and walls [20, 21, 22, 23]. Both fluid migration and secondary diffusion through the air are therefore plausible explanations for VOC detection in nearby comparison samples.

It should be noted that a limitation of these VOC results is the inability to link decomposition VOCs to a specific individual. Moreover, there is currently no broad consensus regarding VOCs that specifically result from human versus animal decomposition. Research on decomposition VOCs remains ongoing, and future findings may help address these limitations.

## 6 LEGAL CONTEXT

A Daubert hearing was conducted on May 15, 2024, to determine the admissibility of this evidence. The judge concluded that HS‐SPME GC–MS for VOC detection meets the standards for scientific validity and admissibility in Indiana courts. This outcome sets a legal precedent for the acceptance of VOC evidence in forensic investigations within the state. A trial was held immediately following this hearing, where the defendant was convicted.

On appeal, the Indiana Court of Appeals affirmed the trial court's evidentiary ruling and upheld the conviction in 2025, rejecting the defendant's challenge to the admission of expert testimony that identified decomposition VOCs in the basement concrete. In its opinion, the court found no reversible error in admitting this scientific evidence.

## 7 CONCLUSION

The identification of multiple characteristic VOCs provides substantial evidence supporting the occurrence of prolonged decomposition at the Michigan City crime scene. The wording of the final laboratory report included the following:

Taken together, four items contained nine VOCs that are known to originate from the decomposition process. This leads to the conclusion that the decomposing body of someone (or something) was present in the room for enough time for the decomposition VOCs to collect on, in, and under the floor.

Ultimately, this forensic evidence contributed to the suspect's conviction, underscoring the important role scientifically validated chemical analyses of VOCs can have in homicide investigations.

## CONFLICT OF INTEREST STATEMENT

The authors declare no conflicts of interest.

## Supporting information


**Table S1.** The table lists all compounds that were identified in the items submitted for analysis. These compounds were not identified in any blanks or control samples.

## Data Availability

The data that support the findings of this study are available on request from the corresponding author. The data are not publicly available due to privacy or ethical restrictions.
