# Supplementary material for: The scent of death: A case study for volatile markers of decomposition on a concrete floor
Source: J Forensic Sci. 2026 Jan 28;71(3):1480–7. doi: 10.1111/1556-4029.70271 (PMC13139789; doi:10.1111/1556-4029.70271)
Supplement: Supplementary file 1 — Table S1. The table lists all compounds that were identified in the items submitted for analysis. These compounds were not identified in any blanks or control samples. [file JFO-71-1480-s001.docx]

| Item # | Description | 2,2,4-trimethyl-3-carboxyisopropyl, isobutyl ester pentanoic acid | 1-propanol | 1-butanol | 3-hexen-2-one | Acetone | Octanoic acid | methyl cyclopentane | 1-hexene | 2-pentanone | 2-nonanone | 2-undecanone |
| --- | --- | --- | --- | --- | --- | --- | --- | --- | --- | --- | --- | --- |
| 4 | Control #1 (scraping) |  |  |  |  |  |  |  |  |  |  |  |
| 4 | Control #1 (dry swab) | + |  |  |  |  |  |  |  |  |  |  |
| 4 | Control #1 (wet Swab) | + |  |  |  |  |  |  |  |  |  |  |
| 5 | Control #2 (scraping) |  |  |  |  |  |  |  |  |  |  |  |
| 5 | Control #2 (dry swab) | + |  |  |  |  |  |  |  |  |  |  |
| 5 | Control #2 (wet swab) | + |  |  |  |  |  |  |  |  |  |  |
| 6 | Control #3 (scraping) |  | + | + | + |  |  |  |  |  |  |  |
| 6 | Control #3 (dry swab) | + |  |  |  |  |  |  |  |  |  |  |
| 6 | Control #3 (wet swab) | + |  |  |  |  |  |  |  |  |  |  |
| 8 | Sample #5 (scraping) |  |  |  |  | + |  |  |  |  |  |  |
| 8 | Sample #5 (dry swab) | + |  |  |  |  |  |  |  |  |  |  |
| 8 | Sample #5 (wet swab) | + |  |  |  |  | + |  |  |  |  |  |
| 9 | Sample #6 (scraping) |  |  |  |  |  |  |  |  |  |  |  |
| 9 | Sample #6 (dry swab) |  |  |  |  |  |  |  |  |  |  |  |
| 9 | ample #6 (wet swab) | + |  |  |  |  |  |  | + |  |  |  |
| 10 | Sample #7 (scraping) |  |  |  |  |  |  |  |  |  |  |  |
| 10 | Sample #7 (dry swab) |  |  |  |  |  |  |  |  |  |  |  |
| 10 | Sample #7 (wet swab) | + |  |  |  |  |  |  |  | + | + | + |
| 11 | Sample #8 (scrapings) |  |  |  |  |  |  |  |  |  |  |  |
| 11 | Sample #8 (dry swab) | + |  |  |  |  |  |  |  |  |  |  |
| 11 | Sample #8 (wet swab) | + |  |  |  |  |  | + |  |  |  |  |
